# Quantifying the volume of nitrous oxide mix wasted through stock rotation in a manifold supply system

**DOI:** 10.1002/anr3.70086

**Published:** 2026-07-09

**Authors:** J. Waller, J. Russell, E. Harman, C. Mitchell, C. El Zerbi, C. Hadley, C. Shelton

**Affiliations:** ^1^ School of Medicine and Population Health The University of Sheffield Sheffield UK; ^2^ Lancaster Medical School, Faculty of Health and Medicine Lancaster University Lancaster UK; ^3^ North West School of Anaesthesia NHS England Workforce Training and Education North West Manchester UK; ^4^ Department of Pharmacy, Wythenshawe Hospital Manchester University NHS Foundation Trust Manchester UK; ^5^ Department of Health Research, Faculty of Health and Medicine Lancaster University Lancaster UK; ^6^ Department of Anaesthesia, Wythenshawe Hospital Manchester University NHS Foundation Trust Manchester UK

**Keywords:** Anesthetics, Inhalation, Delivery of Health Care, Global Warming, Greenhouse Gases, Nitrous oxide

## Abstract

Nitrous oxide accounts for more healthcare‐associated carbon‐equivalent emissions than all other anaesthetic gases combined. Prior research has demonstrated significant waste of pure nitrous oxide. However, waste from nitrous oxide mix (‘gas and air’) remains poorly characterised. We conducted a single‐site service evaluation of waste due to returns of unused nitrous oxide mix. A manifold‐based system of eight G‐size cylinders was assessed across four exchanges in two non‐consecutive weeks. Cylinders were weighed following removal from the manifold. The residual mass of gas was calculated by subtracting the tare weight from the total mass. Results were analysed using descriptive statistics. Carbon‐equivalent greenhouse gas emissions were estimated using 100‐year global warming potential (273). The mean residual mass per cylinder was 1.25 kg (SD 0.53; range 0–2.1), representing 14.8% of original contents. Across four exchanges, 38.9 kg of nitrous oxide mix was returned unused, equivalent to 6.17 tonnes CO_2_. Waste is embedded in the routine practice of maintaining an uninterrupted supply of nitrous oxide mix. There is substantial variance, both between and within cylinder bank changes, which may be due to leaks or gas handling practices. Multi‐site evaluations are needed to develop a broader understanding of waste, and why it occurs.

## Introduction

Reducing the atmospheric emissions of nitrous oxide is arguably the highest‐impact intervention in reducing the environmental impact of anaesthetic practice [1]. As a greenhouse gas, it has an atmospheric lifespan of 115 years and 100‐year global warming potential (GWP_100_) 273 times that of carbon dioxide (CO_2_) [2]. The atmospheric concentration of nitrous oxide is four orders of magnitude lower than that of CO_2_, yet it accounts for approximately 7% of effective radiative forcing (the process that leads to global warming) [2]. Because of its high GWP_100_ and volume of procurement in the National Health Service (NHS), nitrous oxide accounts for more greenhouse gas emissions than all other anaesthetic gases [3].

Work to minimise the environmental impact of nitrous oxide is already underway. The Nitrous Oxide Project demonstrated significant wastage in centralised (pipeline and manifold) supply systems of pure nitrous oxide [1]. Consequently, many NHS Trusts across the UK have transitioned to using point‐of‐care cylinders in the operating theatre to minimise waste without affecting patient care. This approach is supported by a national professional consensus statement [4].

Mitigating the emissions of nitrous oxide mix (often known as ‘gas and air’, or by the brand names Entonox® and Equanox®) is more complex, and it currently accounts for nearly three‐quarters of healthcare‐related nitrous oxide emissions in England [5]. Although approaches to waste mitigation for nitrous oxide mix have been suggested, no clear guidance exists. It is widely used for labour analgesia and has roles in other areas, such as endoscopy and emergency departments [6, 7]. While its analgesic efficacy has been found to be rather limited in comparison to neuraxial analgesia [8], nitrous oxide mix remains the most common labour analgesic in the UK, and its availability is recommended by the National Institute for Health and Care Excellence (NICE) [9]. Consequently, it is likely to remain a mainstay of labour analgesia for the foreseeable future.

Minimising wastage is essential to reducing environmental impact, but the problems must be understood before measures can be implemented. Drawing on experiences with piped pure nitrous oxide, pre‐utilisation losses from system leaks, procurement and storage practices are important candidates for consideration [1].

While there are no definitive data at present, a pilot study of nitrous oxide mix procurement conducted by our group showed significant variation between NHS Trusts in England [10], indicating possible over‐procurement or pre‐utilisation loss due to leaks. These insights illustrate the need to develop empirical evidence by quantifying the volume of nitrous oxide mix left in returned cylinders.

Waste from over‐procurement and returns is important. According to professional guidance, part‐full cylinders must be emptied before refilling to prevent contamination and/or inaccurate gas mix composition [11]. This means that all nitrous oxide mix procured by NHS Trusts is ultimately lost to the atmosphere, whether it is used or not.

Here, we report a service evaluation which aimed to explore stock‐rotation losses from a nitrous oxide mix manifold at a single NHS maternity service. It was conducted both as a basis for local quality improvement and to build understanding in anticipation of a wider project. It is reported in accordance with relevant Sustainability in Quality Improvement (SusQI) templates [12].

## Methods

This service evaluation investigated nitrous oxide mix wastage through stock rotation at the maternity nitrous oxide mix manifold at Wythenshawe Hospital, a large teaching hospital where approximately 5000 babies are born annually. The manifold unit, adjacent to the hospital's maternity department, contains two banks of eight G‐size cylinders, each with an internal volume of 23.6 l. When full, the cylinders contain 5000 l of gas (at standard temperature and pressure), compressed to 137 bar.

One bank is active at a time, with all eight cylinders connected to a manifold via pin‐index valves. Once the pressure in the active bank falls to 13 bar, the supply switches over to the other bank, prompted by the activation of an alarm. At 13 bar, approximately 9.5% of the original gas remains. The manifold room also stores newly delivered cylinders which have not yet been attached, and used cylinders awaiting collection by the manufacturer, which typically takes place on Monday and Friday each week (an additional collection on a Wednesday is possible, if needed).

Each gas in the nitrous oxide mix is 50% by volume, meaning that there are the same number of moles of nitrous oxide and oxygen (111.6 in a G‐size cylinder, based on each mole occupying 22.4 l at standard temperature and pressure). Based on their molecular masses (nitrous oxide 44; oxygen 32) it can be calculated that there are 3.571 kg of oxygen (42.1%) and 4.911 kg of nitrous oxide (57.9%) in a full cylinder (total mass 8.482 kg).

With appropriate permissions from our institutional Pharmacy and Estates Departments and Medical Gases Group, we conducted four spot checks of cylinder changes. These took place on two different weekdays on two non‐consecutive weeks, with the aim of quantifying nitrous oxide mix wastage. Cylinders were weighed before return to the manufacturer using an industrial scale with a precision of ± 100 g (DT Drive Thru Platform Scales, Marsden Weighing Machine Group Ltd, Rotherham, UK). The tare weight (indicating the mass of the empty cylinder including the valve) was subtracted from the total mass recorded by the scales, leaving the total mass of gas remaining in the cylinder.

Results were recorded in a spreadsheet (Excel, Microsoft Corporation, Redmond, Washington, USA) and analysed using descriptive statistics. The carbon footprint of retained gases was calculated as carbon dioxide equivalents (CO_2_e) based on the GWP_100_ value of 273 from the Intergovernmental Panel on Climate Change *Sixth Assessment Report* (IPCC AR6) [2], using the formula CO_2_e = mass of N_2_O × GWP_100_.

## Results

Measurements were taken on 27 November 2025 and 2 December 2025 (week 1, exchanges 1 and 2), and 22 January 2026 and 26 January 2026 (week 2, exchanges 1 and 2).

**Figure 1:**
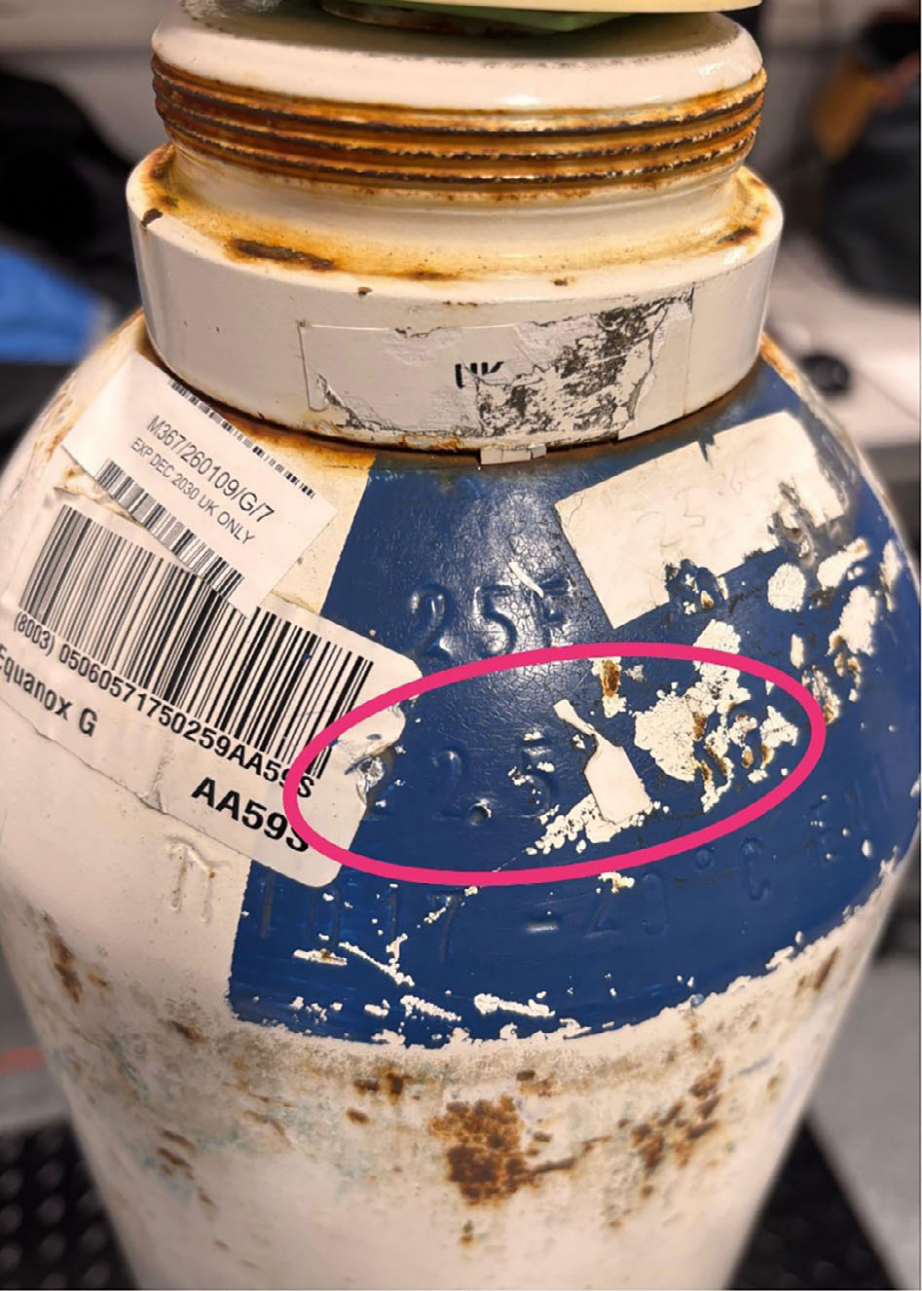
Representative photograph of the tare weight (22.5 kg; indicated by the magenta ellipse) debossed on the shoulder of a G‐size nitrous oxide mix cylinder, amongst other markings and stickers. This illustrates a potential challenge of this approach.

Cylinder tare weights debossed on the cylinder shoulders ranged from 22.4 kg to 34.7 kg. They proved difficult to find in many cases, often being concealed behind stickers or obscured by paint (see Fig. 1). The tare weight for one cylinder in week 1 exchange 1 was unidentifiable, resulting in the exclusion of the cylinder from the results.

For both weeks, exchange 1 produced lower percentages of gas returned to the supplier (14.5% and 10.9% for weeks 1 and 2, respectively) in comparison to exchange 2 (16.9% and 16.8%) (Table 1).

**Table 1:** Nitrous oxide mix return data.

Week	Exchange	Mean mass of N_2_O mix returned per cylinder*	Lowest mass of N_2_O mix returned per cylinder	Highest mass of N_2_O mix returned per cylinder	Total mass of N_2_O mix returned	Total mass of N_2_O returned	Total CO_2_e of N_2_O returned
1	1*	1.2 kg	0 kg	1.7 kg	8.6 kg	5.0 kg	1365 kg
	2	1.4 kg	0.5 kg	2.1 kg	11.5 kg	6.7 kg	1829 kg
2	1	0.9 kg	0.4 kg	1.8 kg	7.4 kg	4.3 kg	1174 kg
	2	1.4 kg	0.5 kg	1.8 kg	11.4 kg	6.6 kg	1802 kg
**Two‐week totals**					38.9 kg	22.6 kg	6170 kg

* Based on eight cylinders, except in week 1 exchange 1, where one cylinder was excluded because no tare weight could be identified.

The mean residual gas per cylinder was 1.25 kg (SD 0.53; range 0–2.1 kg), representing 14.8% of original contents. This exceeded what would be expected based on manifold switching at 13 bar (9.5%).

Each week, 16 cylinders containing a total of 135.7 kg of nitrous oxide mix are supplied. Of this, nitrous oxide makes up 78.6 kg, leading to greenhouse gas emissions of 21.45 t of CO_2_e weekly. Of this, an average of 3.22 t CO_2_e is wasted through returns.

Based on the mean and SD of our data, we calculate a 95% confidence interval of between 1.06 and 1.44 kg of retained nitrous oxide per cylinder. Multiplying this by the 832 nitrous oxide mix cylinders supplied annually (16 cylinders x 52 weeks) gives a speculative estimate of between 882 and 1198 kg of nitrous oxide mix returned per year, equivalent to between 139.42 and 189.36 t of CO_2_e.

## Discussion

These snapshot data indicate that 14.8% of the nitrous oxide mix procured for use at the maternity manifold at our institution is wasted through stock returns. This exceeds the percentage that would be predicted based on the operation of the manifold system (9.5%). Given that the schedule of gas deliveries is relatively rigid, it is likely that this is due to the pragmatic management of the system, by switching banks before the pressure falls to 13 bar to facilitate scheduled replacement of the part‐empty cylinders. The process of integrating manifold management practices with the scheduling of gas delivery may also explain why there was a higher proportion of retained gas on the second exchange in both weeks.

We noted a wide variation in the mass of retained nitrous oxide mix, even within the same exchange cycle. For example, in week 1 exchange 2, there was a range of 1.6 kg between the lowest (0.3 kg) and highest (2.1 kg) masses of retained gas. The reasons for this are not clear, but potential explanations include leaks, for example, while attached to the manifold due to an inadequate Bodok seal or following removal due to inadequate closure of the pin‐index valve; or variations in emptying due to orifice restrictions or temperature variations (e.g. due to the Joule‐Thompson effect [13]).

Another key finding is that a degree of waste is always embedded in the current practice of changing medical gas cylinders. The design of the manifold system dictates when the routine process of exchange takes place, and this is governed by the overarching need to ensure a continuous supply. In our institution, this routine process is triggered by an alarm which sounds at 13 bar, when 9.5% of the gas remains. Consequently, a degree of wastage is a systematic outcome of the way the practice is currently configured. However, it is unknown at this point whether this current configuration is optimal or whether a lower alarm threshold could reduce the volume of embedded waste while still maintaining constant availability of nitrous oxide mix. It should be noted that complete depletion of cylinders is not advised – manufacturers suggest that cylinder use should cease at low positive pressure (2 bar) [14], to prevent ingress of contaminants.

This single‐site service evaluation was conducted in part as a pilot exercise to inform the development of a wider project, in recognition that it may be beneficial to gather data at multiple institutions with differing delivery schedules, clinical demands and service configurations. However, we encountered challenges locating the tare weights for the cylinders (Fig. 1), and the need to use a large industrial scale to safely weigh the cylinders also presented some logistical challenges related to installing the scales on a flat surface and then safely moving the cylinders to that location, which required the use of a transport trolley. With this in mind, we plan to revisit the techniques for the measurement of retained gas before collecting further data. In our opinion, basing this on pressure and temperature measurements is likely to be more feasible for routine use, although colleagues elsewhere have used continuous weight assessment to monitor medical gas depletion in the context of pure nitrous oxide, and this approach may be appropriate in some settings [15].

The Nitrous Oxide Project [1] has characterised the scale of waste from piped pure nitrous oxide and this has translated into impactful national guidance which directs organisations to minimise waste without restricting clinical autonomy [10]. This study, together with our previous pilot work which compared national data on procurement of nitrous oxide mix and the size of clinical services [10] contributes to a growing evidence base which suggests that there may also be a significant quantity of waste from nitrous oxide mix, which may likewise be mitigated. Notably, current guidance on reducing waste from nitrous oxide mix encourages the ‘optimisation’ of manifold and pipeline supply systems in high use areas, such as maternity units [16], but does not identify changes to stock replenishment practices as a means to achieve this.

The NHS in England has committed to become ‘carbon net zero’ for direct greenhouse gas emissions by 2040 [17]. At present, nitrous oxide mix is the most significant source of emissions from anaesthetic gases in the NHS (157,297 t CO_2_e in financial year 2024/2025), dwarfing the emissions from all other anaesthetic gases combined (54,486 t CO_2_e from pure nitrous oxide and 13,399 t CO_2_e from volatile agents) [5]. This emphasises the need for further research to build on this work and the previous pilot study [10] and gain a comprehensive understanding of both the amount of waste that occurs within medical gas management systems and how and why it occurs, across maternity services in the United Kingdom. These insights can then inform the development of practical, acceptable and transferable methods to mitigate this loss, while safeguarding the analgesic choices of women in labour.
